# Hospital acquired infections in a private paediatric hospital in Kenya: a retrospective cross-sectional study

**DOI:** 10.11604/pamj.2022.41.28.25820

**Published:** 2022-01-11

**Authors:** Rohini Kalagouda Patil, Beatrice Kabera, Charles Kiilu Muia, Boni Maxime Ale

**Affiliations:** 1 Department of Paediatrics, Gertrude´s Children´s Hospital, Nairobi, Kenya,; 2 Department of Clinical Pathology, Gertrude´s Children´s Hospital, Nairobi, Kenya,; 3 Department of Laboratory Sciences, Gertrude´s Children´s Hospital, Nairobi, Kenya,; 4 Holo Healthcare Limited, Nairobi, Kenya

**Keywords:** Hospital acquired infections, nosocomial infections, children, antimicrobial resistance

## Abstract

**Introduction:**

Hospital acquired infections (HAI) or infections acquired in a hospital setting significantly increase morbidity and mortality, prolong hospital stay and increase healthcare costs. Factors like malnutrition and irrational use of antibiotics in a resource limited setting contribute to poor outcome in children. Thus a retrospective cross-sectional study was undertaken to study the different types of HAI in children, the different organisms causing them and their sensitivity to different antimicrobials so as to inform appropriate empirical antimicrobial therapy initiation and thus prevent antimicrobial resistance in the region.

**Methods:**

children aged one day to eighteen years, admitted to the hospital for at least 48 hours, during the period of January 2015 to December 2016, with positive laboratory findings on clinical specimens and clinical features in keeping with HAI were included.

**Results:**

the total number of HAI were fifty-two infections in forty-one cases of which, twenty-five cases were culture proven bacterial HAI. Six cases had more than one HAI. The point prevalence of culture positive bacterial HAI in this study was 2.62% (95%CI: 3.8-6.7). The gastrointestinal infections (53%), blood stream infections (21%), lower respiratory tract infections (11%) were the commonest hospital acquired infections. Klebsiella Pneumoniae was the most common bacteria causing HAI with 61.53% of multidrug resistance strains.

**Conclusion:**

gastrointestinal infections were the commonest HAI followed by blood stream infections. The commonest bacteria causing HAI was Klebsiella pneumoniae. The multidrug resistant organisms were Klebsiella Pneumoniae, Enterobacter Cloacae and Acinetobacter baumannii mainly resistant to third and fourth generation cephalosporins and carbapenems.

## Introduction

Nosocomial or hospital acquired infections (HAI) are those that are acquired in a hospital setting. The factors responsible for this are multiple and include host factors such as greater severity of illness, relative immunocompromised state, prior administration of antibiotics and treatment related factors necessitating the use of invasive devices and invasive procedures. Nosocomial infections significantly increase morbidity and mortality, prolong hospital stay and increase healthcare costs directly and indirectly. The incidence of nosocomial infections ranges from 2.8% to 21.6% [1-5]. They are likely to be more frequent and serious in developing countries, with possible risk factors being malnutrition, delayed presentation to referral centres and multi-organ involvement at admission [6-13]. The resource limited settings combined with these nosocomial infections, lead to a poor outcome [14]. The irrational use of antibiotics compounds to the existing problems. The indiscreet and rampant use of powerful empirical antimicrobial agents is very harmful as it fosters resistance among organisms and favours an increase in nosocomial infections. To reduce these compounding problems, intensive care units are required to maintain an ongoing surveillance for early detection of nosocomial infections, rapidly identify the organisms responsible and initiate appropriate antimicrobial agents based on the prevailing sensitivity patterns.

In sub-Saharan Africa, paediatric nosocomial infections impose a major health burden. The World health organization (WHO) patient safety programme did a systematic review of health care associated infections in developing countries between 1995 and 2008 and found no reports about nosocomial bacteraemia in adults or children in Africa and only 6 studies of paediatric nosocomial bacteraemia from developing countries worldwide [15]. However the most recent regional study done in 2015, in a private hospital in Kenya looked at spectrum of antimicrobial agents and resistance patterns in all age groups [16]. Thus, a more comprehensive study on nosocomial infections in pediatric age group is warranted. This study was undertaken to analyse the status of nosocomial infections in a private paediatric hospital set-up. The study objectives are first of all to document the common HAIs and the organisms causing them in our setup and secondly to describe the specific antibiotic sensitivity patterns among the bacteria causing HAIs in order to enhance the initiation of appropriate antimicrobial use and prevent irrational use of antibiotics, thus reducing the glaring problem of antimicrobial resistance in the region.

## Methods

**Design:** this was a retrospective cross-sectional study conducted from January 2015 to December 2016

**Setting:** Gertrude´s Children´s Hospital was founded in 1947 and provides services to children coming from all over Kenya, East and Central Africa. The Hospital attends to over 350,000 children as outpatient each year and admits more than 10,000 children as inpatient each year. The general paediatric ward receives children with various diseases, paediatric surgical ward receives all kinds of pre and postoperative surgical cases and Intensive care unit and high dependency units admit critically sick children. The data used in this study was retrieved from three sources of information which includes: clinical microbiology register, individual electronic patient records, and radiological records. All admissions to the intensive care unit, pediatric general wards and post-surgical wards during the period of January 2015 to December 2016; with a positive culture report on various samples confirmed by the laboratory were studied in detail. We selected five main HAIs: blood stream infections (associated with or without central venous catheter), lower respiratory tract infections, urinary tract infections (UTI) with or without a catheter in situ, post-surgical wound infections and gastrointestinal tract infections.

### Participants

**Inclusion criteria:** any child aged one day of life to eighteen years, admitted in Paediatric intensive care unit (PICU), medical and surgical wards for at least 48 hours with any of the following were included in the study. 1) Features of septicaemia with at least one positive blood culture associated with or without central venous catheterization obtained after 48 hours after hospitalization. 2) Symptoms consistent with lower respiratory tract infections with new sputum production with progressive new infiltrate not present on admission chest radiograph with positive tracheal aspirates or positive sputum cultures after 48 hrs of admission. 3) Symptoms consistent with urinary tract infection with or without urinary catheterization with positive urine cultures. 4) Patients who underwent surgery and had pus at incision site within 30 days of surgery or deep surgical wound infections <30 days after surgery accompanied by fever. 5) Diarrhoea with stool/rectal swabs positive for bacterial cultures, or antigen testing for rotavirus and adenovirus, or blood samples positive for *Clostridium difficile* toxins after 2-3 days of admission.

**Exclusion criteria:** children with any of the following were excluded from the study. 1) Children with HAI acquired from other hospitals at the time of admission. 2) Suspected colonization of organisms established after the file reviews.

**Operational definitions:** any infection was considered to be HAI if there was no evidence suggesting that the infection was present or incubating at the time of admission but became clinically evident after 48 hours of hospitalization. HAIs were defined in agreement with the Centre for Disease Control and Preventions definitions [17]. 1) A case with positive blood culture with or without central venous catheterization obtained from a patient after 48 hours of hospitalization and with clinical features of septicaemia was recorded as hospital acquired blood stream infections. 2) A case with positive culture for sputum or tracheal aspirates obtained after 48 hours of admission with symptoms consistent with lower respiratory tract infections and new sputum production with progressive new infiltrate not present on admission chest radiograph was recorded as hospital acquired lower respiratory tract infections. 3) A case with positive urine culture and with symptoms consistent with urinary tract infection with or without urinary catheterisation developing after 48 hours of admission was recorded as a hospital acquired urinary tract infection. 4) A case with a positive culture from the swab taken from a surgical incision site/wound, who underwent surgery and had pus at the incision site within 30 days of surgery or deep surgical wound infections <30 days after surgery accompanied by fever were recorded as post-surgical wound infections. 5) A case with history of diarrhoea and vomiting developing after 3 days of admission with a positive stool culture or positive for rotavirus/adenovirus antigen or positive for *Clostridium difficile* toxin was recorded as hospital acquired gastrointestinal tract infections.

**Data collection:** data was collected using data abstraction form designed as per the objective of the study. The data was collected in three steps. Step 1: the clinical microbiology register from the laboratory was accessed to retrieve the unique hospital identification number (UHID) for all the cases who had tested positive on blood, urine, sputum and stool cultures. The isolated microorganisms and their antibiotic susceptibility patterns, interpreted according to Clinical and Laboratory Standard Institute (CLSI) guidelines were collected. All cases with positive antigen testing for rotavirus, adenovirus and positive for *Clostridium difficile* toxins were also retrieved from the register. Step 2: with the help of the UHID number, each of these cases were studied in detail from the electronic medical records. All cases that met the above operational definition of HAI were selected for the study. Step 3: information regarding the clinical presentation, diagnosis at admission, history of prior antibiotic use, duration of treatment, risk factors (age, immunocompromised state, malnutrition, blood transfusion, multi-organ involvement, invasive devices and invasive procedures), investigations including chest radiographs, mortality rate and length of stay was recorded. The data collected was entered into Epidata 3.1.

**Outcomes of interest:** the primary outcome of interest was the prevalence of culture positive HAI in our institution. The secondary outcome of interest was to assess the burden of antimicrobial resistance among the organisms causing HAIs.

### Sample size and statistical analysis

**Sample size:** as we were aiming to have comprehensive information on HAI in our setting, we enrolled all positive culture reports on various samples, positive reports for rotavirus/adenovirus antigen and positive reports for *Clostridium difficile* toxins, confirmed by the laboratory in the hospital during the study period.

**Statistical analysis:** to address the objectives of our study, we conducted mainly a descriptive statistical analysis by summarising participant demographics and overall clinical outcomes. We described the spectrum of organisms causing specific HAI and the antibiotic sensitivity patterns for gram negative and gram positive organisms. We used proportion (percentages) to describe categorical variables; mean (± standard deviation) for continuous variables; and median (interquartile range) for count variables. Analyses in general employed normal theory methods and residual diagnostics evaluated validity of assumptions; where appropriate. We calculated the point prevalence of culture positive bacterial hospital acquired infections using the below formula with corresponding 95% confidence intervals:


$$\text{Prevalence = }\frac{\text{Total number of culture positive bacterial HAI}}{\text{Total number of culture positive samples}}\times 100$$

All statistical analyses were conducted on Stata (Stata Corp V.15.1, Texas, USA).

**Ethical consideration:** in order to ensure confidentiality and privacy of study participants, the data were entered in a secured database with private login access to only study investigators and statistician. All patient data were anonymised using the UHID number in the Electronic Health Records within the hospital. The study protocol was reviewed and approved by the institutional review board at Gertrude's children hospital (Reference number: GCH051/2020) including the waiver of informed consent before the implementation of the study.

## Results

The general patient demographics and clinical outcomes are summarised in Table 1. The major risk factors in this study were history of prior antibiotic use, invasive devices, multi-organ involvement, blood transfusions and protein energy malnutrition. The total number of admissions for the year 2015 and 2016 were 16,829 cases. The total number of cultures grown in the laboratory between the year 2015 and 2016 were 952 cases. The total number of HAIs in the year 2015-2016 was 52 infections in 41 cases. Six cases had more than one HAI. Out of this 52 HAI, only 25 cases were culture proven bacterial HAIs. The point prevalence of culture positive bacterial HAI in this study was 2.62% (95% CI: 3.8-6.7).

**Table 1:** demographic characteristics and clinical outcomes of study participants, recruited at Gertrude's Children's Hospital in Nairobi (Kenya), from January 2015 to December 2016 (N=41)

Characteristics	Number	%
Gender		
Female	19	46.3
Male	23	56.0
Case fatality rate	6	14.6
Immuno compromised	1	2.4
Prior antibiotic use	35	85.3
Invasive device	23	56.0
Multi-organ involvement	22	53.6
Transfusion	23	56.0
Protein energy malnutrition		
Grade I	1	2.4
Grade II	5	12.1
Grade III	36	87.8
Median (IQR) duration of antibiotic (days)	14(7-21)	
Median (IQR) duration of hospitalization (days)	18 (7-42)	
Median (IQR) age (days)	716 (153-1460)	

**Figure 1:**
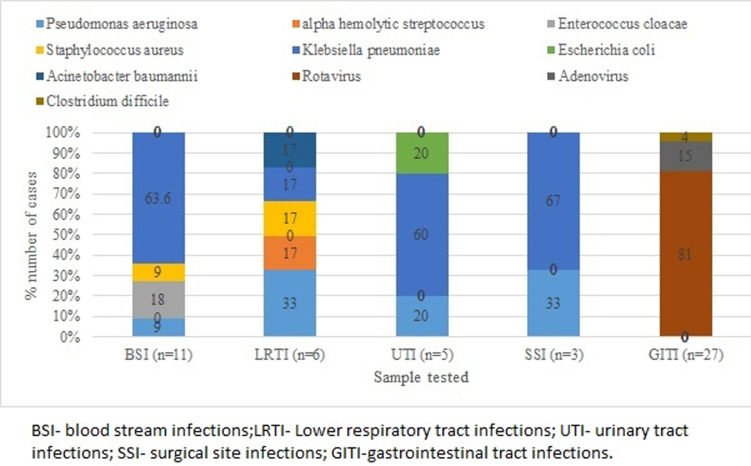
spectrum of organisms causing specific hospital acquired infections in study participants, recruited at Gertrude's Children’s Hospital in Nairobi (Kenya), from January 2015 to December 2016

The hospital acquired infections were gastrointestinal tract infections (53%), blood stream infections (21%), respiratory tract infections (11%), urinary tract infections (9%) and surgical site infections (6%). Thus gastrointestinal tract infections were the most common HAI in this study followed by blood stream infections and respiratory tract infections. The spectrum of organisms causing specific HAI is as shown in the graph (Figure 1). Gastrointestinal tract infections (27) among which rotavirus were 22 (81.48%), Adenovirus were 4 (14.8%), *Clostridium difficile* toxins were positive in 1 (3.70%) case. There were 11 cases of blood stream infections, among which *Klebsiella pneumoniae*, 4 (36.36%), *Enterococcus cloacae* 2 (18.18%) and *Pseudomonas aeruginosa* 1 (9.09%). Catheter related blood stream infections were caused by *staphylococcus aureus* 1 (9.09%) and *Klebsiella pneumoniae* 3 (27.27%). There were 6 cases of lower respiratory tract infections with 1(16.6%) infection caused by *Klebsiella pneumoniae*, 2 (33.33%) caused of *Pseudomonas aeruginosa*, 1 (16.6%) each by *Acinetobacter baumannii, Staphylococcus aureus* and alpha haemolytic *streptococcus* respectively. Urinary tract infections (5 infections) were *Klebsiella pneumoniae* 3 (60%), *Pseudomonas aeruginosa* 1 (20%) and *Escherichia coli* 1 (20%). Surgical site infections (3 infections) were *Pseudomonas aeruginosa* 1 (33.3%) and *Klebsiella pneumoniae* 2 (66.6%).

Rotavirus was the commonest organism with 22 infections (42.3%) of hospital acquired infections followed by *Klebsiella pneumoniae* with 13 infections (25%). *Klebsiella pneumoniae* was the commonest bacterial pathogen contributing to all HAI. The other micro-organisms were *Pseudomonas aeruginosa* with 6 infections (9.6%), adenovirus with 4 infections (7.6%), *Enterobacter cloacae* and *Staphylococcus aureus* with 2 infections each (3.8%). Alpha haemolytic streptococcus, *Escherichia coli, Clostridium difficile* and *Acinetobacter baumannii* caused one infection each (1.9%).

The antibiotic sensitivity patterns for Gram negative and Gram positive organisms is as depicted in Table 2 and Table 3. The bacterial HAI were mainly caused by Gram negative than Gram positive organisms. Of the thirteen (13) infections caused by *Klebsiella Pneumoniae*, 8 (61.5%) were resistant to cephalosporin mainly cefuroxime and cefotaxime. An average of 2 (18.2%) were resistant to ciprofloxacin, meropenem, piperacillin-tazobactum and amikacin. In total, 8 (61.53%) *Klebsiella pneumoniae* species were multidrug resistant. Out of the five infections caused by *Pseudomonas aeruginosa*, 3 (60%) were resistant to Cefotaxime and Ceftriaxone, 1 (16.66%) each was resistant to Cefepime and Piperacillin-Tazobactum. There was one infection caused by multidrug resistant *Acinetobacter baumannii*. The two *Enterobacter cloacae* were resistant to the Cephalosporins and Amoxicillin-clavulanic acid. The two *Staphylococcus aureus* in the study were Cloxacillin sensitive but one was Clindamycin resistant.

**Table 2:** antibiotic sensitivity patterns in gram negative organisms of study participants, recruited at Gertrude's Children's Hospital in Nairobi (Kenya), from January 2015 to December 2016

Organism	total tested	type	gentamycin	trimethoprim/sulfamethoxazole	amoxicillin/clavulanic acid	cefuroxime	cefotaxime	ceftriaxone	ceftazidime	amikacin	ciprofloxacin	meropenem	cefepime	piperacillin	piperacillin /tazobactum	nitrofurantoin	ampicillin sulbactum	ampicillin
Klebsiella pneumoniae**	13	nR	7	7	6	8	8	5	2	1	2	2	1	0	3	1	0	0
		%R	53.8	53.8	46.2	61.5*	61.5*	38.5*	15.4*	7.7*	15.4*	15.4*	7.7*	0	23.1*	7.7*	0	0
		nS	4	6	6	4	4	0	0	8	10	8	0	0	2	2	0	0
		%S	30.8	46.2	46.2	30.8	30.8	0	0	61.5***	76.9***	61.5***	0	0	15.4***	15.4**	0	0
Pseudomonas aeruginosa**	5	nR	0	0	0	0	3	3	0	0	0	0	1	0	1	0	0	0
		%R	0	0	0	0	60*	60*	0	0	0	0	20*	0	20*	0	0	0
		nS	5	0	0	0	0	0	3	5	5	5	4	0	4	0	0	0
		%S	100	0	0	0	0	0	60	100	100	100	80	0	80	0	0	0
Escherichia coli**	1	nR	0	1	1	0	1	0	0	0	0	0	0	0	0	0	0	1
		%R	0	100*	100*	0	100*	0	0	0	0	0	0	0	0	0	0	100*
		nS	0	0	0	1	0	0	0	0	0	0	0	0	0	1	0	0
		%S	0	0	0	100	0	0	0	0	0	0	0	0	0	100	0	0
Enterobact-er cloacae**	2	nR	1	2	1	2	1	1	1	0	0	0	0	0	0	0	0	0
		%R	50	100	50	100	50	50	50	0	0	0	0	0	0	0	0	0
		nS	1	0	0	0	1	0	1	2	2	2	1	1	0	0	0	0
		%S	50	0	0	0	50	0	50	100	100	100	50	50	0	0	0	0
Acinetobac-ter baumannii**	1	nR	1	0	0	1	1	1	1	0	1	0	0	0	1	0	1	0
		%R	100*	0	0	100*	100*	100*	100*	0	100*	0	0	0	100*	0	100*	0
		nS	0	1	0	0	0	0	0	1	0	0	0	0	0	0	0	0
		%S	0	100	0	0	0	0	0	100	0	0	0	0	0	0	0	0

* Resistance; ** Gram negative organism; *** Sensitivity

**Table 3:** antibiotic sensitivity patterns in gram positive organisms of study participants, recruited at Gertrude's Children's Hospital in Nairobi (Kenya), from January 2015 to December 2016

Organism	total tested	type	penicillin	ampicillin	cloxacillin	eythromycin	gentamycin	clindamycin	trimethoprim/sulfamethoxazole	cefotaxime	ceftriaxone	vancomycin	cefuroxime	ceftazidime	cefepime	piperacillin	ciprofloxacin	amikacin	meropenem
Staphylococcus aureus**	2	nR	2	2	0	1	0	1	2	0	0	0	0	0	0	0	0	0	0
		%R	100*	100*	0	50	0	50*	100	0	0	0	0	0	0	0	0	0	0
		nS	0	0	2	1	2	1	0	0	0	0	0	0	0	0	0	0	0
		%S	0	0	100	50	100	50	0	0	0	0	0	0	0	0	0	0	0
Alpha hemolytic streptococc-us**	1	nR	0	0	0	1	0	0	0	0	0	0	0	0	0	0	0	0	0
		%R	0	0	0	100	0	0	0	0	0	0	0	0	0	0	0	0	0
		nS	0	0	0	0	0	1	0	1	1	1	0	0	0	0	0	0	0
		%S	0	0	0	0	0	100	0	100	100	100	0	0	0	0	0	0	0

* Resistance; ** Gram positive organism

## Discussion

The point prevalence of the culture positive HAI in our study was 2.62%. This is much less, than that reported in the developing countries [18], but was comparable to that noted in Algeria [15]. In a HAI surveillance done in three Kenyan hospitals in 2010-2012 showed a prevalence of HAI in paediatric age group was 5.8%, however none of them were bacteriologically confirmed [19]. The gastrointestinal infections were the commonest hospital acquired infections followed by blood stream infections and respiratory tract infections in our study, unlike most other studies where the respiratory tract infections were the commonest hospital infections, followed by blood stream infections and urinary tract infections [20-24]. Although surgical site infection was the commonest HAI reported in the systematic review in sub-Saharan African countries [25], we did not encounter the same. Rotavirus was found to be the major etiologic agent of pediatric nosocomial diarrhoea in our study, similar to that seen in European settings [26].

The three most common bacteria identified in our study were *Klebsiella pneumoniae* followed by Pseudomonas aeruginosa and *Enterobacter cloacae*, which was similar to that seen in other parts of Africa [27,28]. A local study in Kenyatta National Hospital reported *Pseudomonas aeruginosa, Klebsiella pneumoniae, Citrobacter* species and *Staphylococcus aureus* as the most common organisms isolated among the HAI acquired in a mixed intensive care settings [29]. *Klebsiella* (28.5%) followed by *Enterococcus* species (24.4%) were the organisms associated with device associated HAI as seen in most other developing countries [30].

Multiple antibiotic resistance to different classes of antibiotics, including the penicillins, cephalosporins, aminoglycosides, and fluoroquinolones, has been reported to be on the rise amongst most Gram negative pathogens, especially *Klebsiella pneumoniae, Pseudomonas aeruginosa*, and *Acinetobacter baumannii*. Multi-drug resistance organisms pose the greatest threat to infection control measures in the hospital and give rise to frequent hospital outbreaks. In this study, the antimicrobial resistance for *Klebsiella Pneumoniae* was 54.5% which was comparable to the antimicrobial resistance global report on surveillance on African region by World Health Organization (WHO) [31]. Moreover, the resistance of *Klebsiella Pneumoniae* to potent antibiotics like Gentamycin and Ceftriaxone was 54.5% and 38.5% which was comparable to the systematic review of antimicrobial resistance in children in sub-Saharan Africa [32]. Seventy six point nine percent (76.9%) of Klebsiella pneumoniae showed sensitivity to Ciprofloxacin compared to 35-40% sensitivity, and only 15.4% showed sensitivity to Piperacillin -Tazobactum compared to 60% sensitivity in a study from India [33]. *Pseudomonas aeruginosa* was 100% sensitive to ciprofloxacin and meropenem similar to that in India but had 20% resistance to Piperacillin-Tazobactum. Pseudomonas species resistance to Ceftazidime reported in Madagascar was 62% [34], unlike our study which showed 60% sensitivity to Ceftazidime. *Acinetobacter baumannii* was a multidrug resistant organism in this study which was similar to that demonstrated in other developing countries [24,35,36].

Increased antibiotic resistance has been associated with transmission of resistant bacteria within hospitals by cross contamination and colonisation of patients via the hands of healthcare staff and subsequent spread between hospitals by transfer of such colonised patients [37]. Strategies to control antibiotic resistance in hospitals would need multidisciplinary team approach in implementing hospital policies on the use of antibiotics and infection control measures, timely detection and reporting of the antibiotic resistant strains, improved surveillance, and control of transmission of resistant bacteria. Bacterial resistance in device associated infections in six hospitals of Iran showed that 100% of *Staphylococcus aureus* isolates were resistant to oxacillin [36]. The incidence of methicillin resistant *Staphylococcus aureus* in our setting appears to be low.

The limitations of this study were first of all a positive culture was an essential criterion for our study so the actual prevalence of HAI could have been much higher and there was a high likely hood of missing the infections caused by organisms that failed to grow in the laboratory. Secondly, not all viral HAIs could be identified due to limitations in the availability for the appropriate tests. All the data for patient related information was collected from an electronic based patient data system which contained all the information the study intended to collect, however considering the retrospective nature of the study design, a prospective study design would provide more information and aid in HAI surveillance in the hospital.

## Conclusion

In summary, HAI were common in our setup with gastrointestinal infections as the commonest followed by blood stream infections. Thus, the infection control priorities in our hospital should focus in controlling and preventing them. Moreover, the multidrug resistant organisms in our setting (*Klebsiella Pneumoniae, Enterobacter Cloacae* and *Acinetobacter baumannii* mainly resistant to third and fourth generation cephalosporins and carbapenems) gave us an idea about the antibiotic resistance among organisms causing HAI in our region. These findings will form a basis for HAI surveillance and will aid in the antimicrobial stewardship program in our institution and other paediatrics hospitals in the country to enhance the initiation of appropriate antimicrobials

**Funding:** none of the authors received any funding from the Gertrude's Children's Hospital research fund for their contribution to the study.

### What is known about this topic

- Hospital acquired infections or healthcare associated infections significantly increase morbidity and mortality, prolongs hospital stay and increases healthcare costs directly and indirectly;
- Hospital acquired infections together with resource limited settings lead to poor outcomes in children;
- Empirical antimicrobial therapy for suspected hospital acquired infection in a particular region must be guided by the antimicrobial sensitivity and resistance patterns of the organisms isolated from the region or location.

### What this study adds

- Hospital acquired infections or healthcare associated infections significantly increase morbidity and mortality, prolongs hospital stay and increases healthcare costs directly and indirectly;
- Hospital acquired infections together with resource limited settings lead to poor outcomes in children;
- Empirical antimicrobial therapy for suspected hospital acquired infection in a particular region must be guided by the antimicrobial sensitivity and resistance patterns of the organisms isolated from the region or location.

## References

[1] Daschner F (1985). Nosocomial infections in intensive care units. Intensive care Med.

[2] Correia M, Simao C, Lito LM, Cabecadas M, Almeida H, Carvalho A (1997). Nosocomial infections in a paediatric intensive care unit. Acta Med Port.

[3] Donowitz LG (1986). High risk of nosocomial infections in the Pediatric critical care patient. Crit care Med.

[4] Milliken J, Tait GA, Ford Jones JL, Mindorff CM, Gold R, Mullins G (1988). Nosocomial infection in a pediatric intensive care unit. Crit Care Med.

[5] Legras A, Robert R (1999). Nosocomial infection prospective survey of incidence in 5 French ICU. Intensive Care Medicines.

[6] Aiken AM, Mturi N, Njuguna P, Mohammed S, Berkley JA, Mwangi I (2011). Risk and causes of paediatric hospital acquired bacteremia in Kilifi District Hospital, Kenya: a prospective cohort study. Lancet.

[7] Richards MJ, Edwards JR, Culver DH, Gaynes RP (1999). Nosocomial infections in pediatric intensive care units in the United States. National Nosocomial Infections Surveillance System. Paediatrics.

[8] Raymond J, Aujard Y (2000). Nosocomial infections in Pediatric patients: a European, multicentre prospective study. Infect Control Hosp Epidemiol.

[9] Taylor RW, Manganaro L, O´Brien J, Trottier SJ, Parkar N, Veremakis C (2002). Impact of allogenic packed red blood cell transfusion on nosocomial infection rates in the critically ill patient. Crit Care Med.

[10] Du Moulin G (1989). Minimizing the potential for nosocomial pneumonia: architectural engineering and environmental considerations for the intensive care unit. Eur J Clin Microbiol Infect Dis.

[11] B Renaud B, Brun-Bussisson C, ICU-Bacteremia Study Group (2001). Outcomes of primary and catheter-related bacteremia. Am J Respir Crit Care Med.

[12] Shafazand S, Weinacker AB (2002). Blood culture in the critical care unit. Improving utilization and yield. Chest.

[13] Furfaro S, Gauthier M, Lacroix J, Nadeau D, Lafleur L, Mathews S (1991). Arterial catheter-related infections in children. A 1-year cohort analysis. Am J Dis Child.

[14] The global antibiotic resistance partnership-Kenya National Working Group 2011 Situation analysis and recommendations: antibiotic use and resistance in Kenya, executive summary.

[15] Bagheri Nejad S, Allegranzi B, Syed SB, Ellis B, Pittet D (2011). Health-care-associated infection in Africa: a systematic review. Bull World Health Organ.

[16] Daniel M, Geoffrey O, Gunturu R, Adam RD (2016). Spectrum of microbial diseases and resistance patterns at a private teaching hospital in Kenya: implications for clinical practice. PLOS ONE.

[17] Garner JS,Jarvir WR, Emori TG, Horan TC, Hughes JM (1988). CDC definitions for nosocomial infections. Am J Infect Control.

[18] Deptula A, Trejnowska E, Dubiel G, Zukowski M, Misiewska-Kaczur A, Ozorowski T (2017). Prevalence of healthcare-associated infections in Polish adult intensive care units: summary data from the ECDC European Point Prevalence Survey of Hospital-associated Infections and Antimicrobial Use in Poland 2012-2014. Journal of Hospital Infection.

[19] Ndegwa L (2015). Hospital-acquired infections surveillance in three Kenyan Hospitals 2010-2012. Open Forum Infectious Diseases.

[20] Metsini A, Vazquez M, Sommerstein R, Marschall J, Voide C, Troillet N (2018). Point prevalence of healthcare-associated infections and antibiotic use in three large Swiss acute-care hospitals. Swiss Med Wkly.

[21] Chen Y, Zhao JY, Shan X, Han XL, Tian SG, Chen FY (2017). A point-prevalence survey of healthcare-associated infection in fifty-two Chinese hospital. J Hosp Infect.

[22] Zarb P, Coignard B, Griskeviciene J, Muller A, Vankerckhoven V, Weist K (2012). The European Centre for Disease Prevention and Control (ECDC) pilot point prevalence survey of healthcare-associated infections and antimicrobial use. Euro Surveill.

[23] Cassini A, Plachouras D, Beckman´s T, Abu Sin M, Blank HP, Ducomble T (2016). Burden of six healthcare-associated infections on European Population Health: estimating incidence-based disability-adjusted life years through a population prevalence-based modelling study. PLOS Medicine.

[24] Venkataraman Divatia JV, Ramakrishnan N, Chawla R, Amin P, Gopal P (2018). Multicenter observational study to evaluate epidemiology and resistance patterns of common intensive care unit-infections. Indian J Crit Care Med.

[25] Allegranzi B, Bagheri Nejad S, Combescure C, Graafmans W, Attar H, Donaldson L (2011). Burden of endemic health-care-associated infection in developing countries: systematic review and meta-analysis. Lancet.

[26] Gleizes O, Desselberger (2006). Nosocomial rotavirus infection in European countries: a review of the epidemiology severity and economic burden of hospital-acquired rotavirus disease. Pediatr Infect Dis J.

[27] Kesah CN, Egri-Okwaji MT, Iroha E, Odugbemi TO (2004). Aerobic bacterial nosocomial infections in paediatric surgical patients at a tertiary health institution in Lagos, Nigeria. Niger Postgrad Med J.

[28] Yallew WW, Kumie A, Yehuala FM (2016). Point prevalence of hospital-acquired infections in two teaching hospitals of Amhara region in Ethiopia. Drug Healthc Patient Saf.

[29] Ngumi ZW (2006). Nosocomial infections at Kenyatta National Hospital Intensive-Care Unit in Nairobi, Kenya. Dermatology.

[30] Kumar S, Sen P, Gaind R, Verma PK, Gupta P, Suri PR (2018). Prospective surveillance of device-associated health care-associated infection in an intensive care unit of a tertiary care hospital in New Delhi, India. Am J Infect Control.

[31] Essack SY, Desta AT, Abotsi RE, Agoba EE (2017). Antimicrobial resistance in the WHO African region: current status and roadmap for action. Journal of Public Health.

[32] Williams PCM, Isaacs D, Berkley JA (2018). Antimicrobial resistance among children in sub-Saharan Africa. Lancet Infect Dis.

[33] Gopalakrishnan R, Sureshkumar D (2010). Changing trends in antimicrobial susceptibility and hospital acquired infections over an eight-year period in a tertiary care hospital in relation to introduction of an infection control programme. J Assoc Physicians India.

[34] Randrianirina F, Vaillant L, Ramarokoto CE, Rakotoarijaona A, Andriamanarivo ML, Razafimahandry HC (2010). Antimicrobial resistance in pathogens causing nosocomial infections in surgery and intensive care units of two hospitals in Antananarivo, Madagascar. J Infect Dev Ctries.

[35] Chittawatanarat K, Jaipakdee W, Chotirosniramit N, Chandacham K, Jirapongcharoenlap T (2014). Microbiology, resistance patterns, and risk factors of mortality in ventilator-associated bacterial pneumonia in a Northern Thai tertiary-care university based general surgical intensive care unit. Infect Drug Resist.

[36] Jahani-Sherafat S, Razaghi M, Rosenthal VD, Tajeddin E, Seyedjavadi S, Rashidan M (2015). Device-associated infection rates and bacterial resistance in six academic teaching hospitals of Iran: findings from the International Nocosomial Infection Control Consortium (INICC). J Infect Public Health.

[37] Ducel G, Fabry J, Nicolle L, World Health Organization (2002). Prevention of hospital-acquired infections: a practical guide.

